# Feasibility of Privacy-Preserving LiDAR-Based Continuous Gait and Activity Monitoring in Three People with Multiple Sclerosis: A Technical Proof-of-Concept Case Study

**DOI:** 10.3390/s26144455

**Published:** 2026-07-14

**Authors:** Michael Single, Sara Mollà-Casanova, Lena C. Bruhin, Vasileios Skaramagkas, Stephan M. Gerber, Andrew Chan, Tobias Nef, Iris-Katharina Penner

**Affiliations:** 1 Gerontechnology and Rehabilitation Group, ARTORG Center for Biomedical Engineering Research, University of Bern, 3012 Bern, Switzerland; lena.bruhin@unibe.ch (L.C.B.); vasileios.skaramagkas@unibe.ch (V.S.); stephan.m.gerber@unibe.ch (S.M.G.); tobias.nef@unibe.ch (T.N.); 2 Research Unit in Clinical Biomechanics (UBIC), Department of Physiotherapy, Faculty of Physiotherapy, University of Valencia, 46010 Valencia, Spain; sara.molla@uv.es; 3 Department of Neurology, Inselspital, Bern University Hospital, University of Bern, 3012 Bern, Switzerland; andrew.chan@insel.ch (A.C.); iris-katharina.penner@insel.ch (I.-K.P.)

**Keywords:** multiple sclerosis, LiDAR, contactless monitoring, gait analysis, activities of daily living, motor fatigue, digital measures

## Abstract

Multiple sclerosis (MS) is a chronic central nervous system disease with heterogeneous symptoms, including gait disturbances and motor fatigue, affecting daily functioning and quality of life. Episodic assessments may miss within-day functional fluctuations, whereas home-like monitoring may characterize them. This technical proof-of-concept case study quantified gait parameters (velocity, step length, and variability) during natural walking, explored temporal changes in gait and activity as potentially fatigue-relevant motor-performance patterns, and examined the feasibility of deriving candidate digital measures in MS. Three individuals with MS (one EDSS 1; two EDSS 3) were monitored in an instrumented apartment for 6.5–9.0 h using three LiDAR sensors. Gait parameters, region transitions, activity patterns, EDSS, FSMC, VAS-F, and available data-yield indicators were summarized descriptively. Compared with published healthy-adult references, P01 and P03 showed lower walking velocity, and P03 showed reduced step length. P01 maintained stable gait, P02 increased afternoon walking velocity, and P03 showed an afternoon velocity decline and a smaller step length decrease. The behavioral profiles described different spatial activity patterns, and the activity levels remained low during monitoring. LiDAR-based monitoring may provide a privacy-preserving approach to capture gait and activity variations as candidate variables for future validation without establishing fatigue specificity, clinical validity, or diagnostic thresholds.

## 1. Introduction

Multiple sclerosis (MS) is a chronic disease of the central nervous system and one of the most prominent neurodegenerative disorders. An estimated 2.8 million people live with MS (35.9 per 100,000 population [1]), with Switzerland showing a higher and rising prevalence at approximately 110 per 100,000 individuals [2,3]. MS is characterized by demyelination of nerve fibers in the central nervous system, leading to impaired brain–body connectivity [4]. The symptoms vary substantially among individuals and include motor impairments, such as unsteady gait, tremor, and motor fatigue, as well as non-motor issues, including vision disturbances, sensory deficits, cognitive impairment, depression, and anxiety [5]. Early in the disease course, persistent fatigue commonly compromises the quality of everyday activities.

Fatigue in MS manifests as both state fatigue (transient situational exhaustion) and trait fatigue (chronic persistent exhaustion over extended periods). The former provides critical insights into daily fluctuations and activity limitations, while the latter captures the cumulative burden affecting long-term quality of life [6]. Understanding both dimensions is crucial for developing targeted interventions. By limiting daily activities, employment, and social participation, fatigue can undermine independence and reduce quality of life, sharply contrasting with patients’ strong desire to maintain independent living and professional engagement [7].

The current MS monitoring strategies rely on regular clinical assessments, imaging, and laboratory tests to track disease progression and treatment response [8,9]. While these tools offer disease-specific information, they provide limited insights into how MS affects daily functioning continuously and in ecologically valid contexts. Imaging and laboratory tests do not provide specific insights into activities of daily living (ADLs), which are closely linked to daily functioning and represent promising targets for digital measures for personalized care [10]. ADL performance is particularly affected by motor fatigue, which is common in people with MS (pwMS) and typically worsens throughout the day [11]. Identifying motor fatigue determinants could enable targeted interventions to maintain or adapt ADL performance, thereby improving patients’ quality of life [12]. Neurologists use the Expanded Disability Status Scale (EDSS; [13]) as a standardized clinical tool to classify disease severity and monitor disability progression in pwMS, although such assessments have notable limitations, including non-linearity, overemphasis on ambulation, a lack of sensitivity at higher grades, and exclusion of important non-motor symptoms such as cognitive fatigue [14]. Fatigue-specific self-report instruments, such as the Fatigue Scale for Motor and Cognitive Functions (FSMC) and the Visual Analogue Scale to Evaluate Fatigue Severity (VAS-F), complement disability scoring by assessing motor and cognitive fatigue and subjective fatigue severity [15,16]. These shortcomings underline the need for comprehensive symptom evaluation to personalize treatments.

Despite the recognized importance of ADL monitoring in MS progression, a significant gap exists between the episodic nature of clinical assessments and the fluctuations of MS symptoms. Digital measures derived from continuous monitoring could provide objective ecologically valid indicators of functional capacity outside conventional clinical assessments. However, feasibility studies in home-like instrumented environments represent an intermediate step between supervised laboratory testing and fully free-living home monitoring [17]. Unobtrusive measurement approaches can capture natural behavior without the constraints of clinical settings or the burden of wearables. Recent advances in remote monitoring technologies have shown promise in capturing disease-relevant signals in neurological conditions [18]. Specifically in MS, previous studies have employed accelerometers to detect gait abnormalities, smartphone applications for cognitive assessments, and wearables for fatigue monitoring [19,20,21]. However, many of these approaches remain limited by adherence requirements, user burden, or restricted contextual information, which may reduce their ability to capture ADL-related motor changes and intraday fluctuations that are relevant to motor fatigue.

Several studies have proposed sensor-based tools for evaluating motor fatigue and its associated signs and symptoms, including wearable sensors (e.g., inertial measurement units) and non-wearable systems (e.g., motion capture systems) [19]. Light detection and ranging (LiDAR) is an optical sensing technology that measures distances by emitting infrared laser light and measuring the time required for the reflected signal to return to the sensor. LiDAR-based leg tracking offers distinct advantages, particularly for unobtrusive monitoring and ease of use in home-like settings [22,23]. LiDAR sensors provide direct and non-invasive distance measurements [24]. While video-based motion tracking systems can also estimate positional information, their dependence on vision-based algorithms makes them susceptible to privacy concerns. In contrast, LiDAR-based measurements offer a promising balance between previously evaluated gait-measurement performance, unobtrusiveness, and privacy for monitoring gait in home-like settings as they inherently provide continuous distance measurements without capturing sensitive visual data.

Walking velocity and step or stride length are among the spatiotemporal gait parameters consistently reported as altered in pwMS, while step count provides an interpretable measure of ambulatory activity volume that is relevant to real-world walking behavior [19,25,26].

This pilot proof-of-concept case study investigates gait and ADL-related parameters in three pwMS using privacy-preserving 2D LiDAR sensors within an instrumented home-like apartment, the NeuroTec Loft [27]. As an exploratory technical proof-of-concept study, the small sample size enabled in-depth analysis of individual patterns while providing feasibility and data-yield information for larger-scale investigations. However, the study was not intended to support statistical inference, subgroup comparison, diagnostic accuracy, or clinical validation of fatigue-related outcomes. The study specifically aimed to: (1) quantify gait parameters (velocity, step length, and their variability) during natural walking throughout the day as indicators of ADL performance; (2) descriptively explore temporal changes in gait and activity parameters between morning and afternoon as exploratory potentially fatigue-relevant changes in motor performance; and (3) explore this method’s potential for deriving candidate digital measures that may help to characterize daily functioning in pwMS. We expected that, despite relatively similar disability levels as measured by the EDSS, the participants would exhibit individual differences in gait patterns that are detectable by LiDAR. Accordingly, we anticipated that morning–afternoon differences might be observable, consistent with potential fatigue-related effects on daily functioning. However, these differences were interpreted as descriptive and hypothesis-generating rather than as evidence of MS-specific motor fatigue.

## 2. Materials and Methods

This study was approved by the Ethics Committee of the Canton of Bern, Switzerland (KEK no. 2022-00666, date of approval: 17 June 2022), and conducted in accordance with the latest version of the Declaration of Helsinki. All the participants signed a written informed consent form before participating.

### 2.1. Study Participants

Three pwMS who were able to walk unaided and understand the study instructions were recruited from the MS outpatient population at University Hospital Bern (Switzerland). They did not present with traumatic pathology affecting the legs, other neurologic diseases, alterations of the vestibular system, or other concomitant diseases expected to substantially affect gait or balance. The sample size was selected for an initial technical proof of concept and in-depth case-level description of LiDAR-based monitoring in a home-like environment. The one-day monitoring design also enabled descriptive within-participant comparison of gait and activity estimates between the morning and afternoon, with each participant’s morning values used as an individual reference for later values. This comparison was intended to characterize intraday variation within the monitored session, not to establish fatigue-specific effects or replace a control group. The sample was not intended to support statistical inference, subgroup comparison, estimation of diagnostic accuracy, or clinical validation of fatigue-related outcomes. Because no healthy control group or stratified MS comparison group was included, all analyses were descriptive, and observed individual differences cannot be attributed specifically to MS-related motor fatigue; they may also reflect age, habitual activity patterns, task selection, environmental context, or other individual factors.

### 2.2. Experimental Protocol and LiDAR Sensor Setup

**Figure 1:**
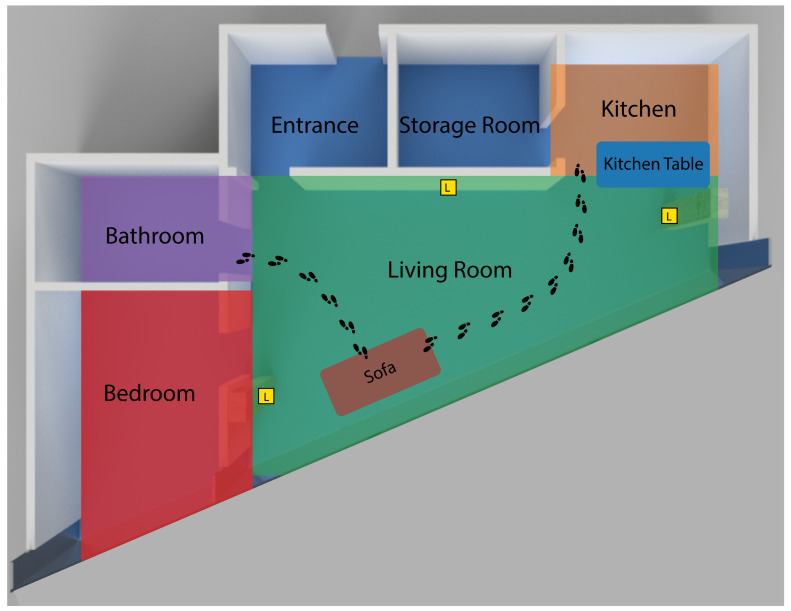
Visualization of the region map in the NeuroTec Loft. Regions in the loft are displayed as color-coded square areas with overlaid annotations of the corresponding region. The installed LiDAR sensors are marked with orange boxes and the capital letter ‘L’. An exemplary walking trajectory is indicated in the floor plan.

The study was conducted in a home-like instrumented apartment, the NeuroTec Loft [27], located at the Swiss Institute for Translational and Entrepreneurial Medicine (SITEM; Inselspital Bern, Switzerland). Three LiDAR sensors (UST-20LX-H01, Hokuyo Automatic Co., Ltd., Osaka, Japan) were installed to span a triangular area encompassing the entire living room and part of the kitchen. The sampling rate of the LiDAR sensors was set to 40 Hz, and the sensors were placed similarly to the setup described by [24]. The sensor arrangement and the labeled regions used for subsequent position assignment are shown in Figure 1.

Upon arrival in the morning, the experiment was verbally explained to the participant. A clinician collected demographic data, including age, sex, and time since first diagnosis. Clinical data such as the EDSS score [13], the Fatigue Scale for Motor and Cognitive Functions (FSMC) score [15], and the Visual Analogue Scale to Evaluate Fatigue Severity (VAS-F) score [16] were assessed prior to the measurement in the loft. Participants were physically alone in the apartment during data collection but were remotely supervised by clinical study staff throughout the procedure for safety and protocol adherence.

Participants stayed in the NeuroTec Loft alone for up to 9 h, starting no earlier than 08:00. During their stay, participants were free to structure their activities independently, while a predefined selection of optional tasks (e.g., cooking a meal, watching a film, or reading a newspaper) was made available. This free-activity design was chosen to evaluate unobtrusive LiDAR monitoring under home-like conditions and to preserve naturalistic behavior. No standardized task schedule, standardized walking protocol, or fatigue-induction procedure was applied. Consequently, differences in activity level or gait over time may reflect task selection, sedentary behavior, motivation, environmental stimulation, or individual habits rather than MS-related motor fatigue. The LiDAR system continuously recorded movement-related data throughout the stay.

### 2.3. LiDAR-Based Leg Tracking

The LiDAR sensors recorded two-dimensional distance scans of the monitored space. As described previously [24], the sensors were based on time-of-flight technology and used a rotating infrared laser to measure distances. The sensors performed scans at 40 Hz, with each scan covering a 270-degree field of view at an angular resolution of 0.125 degrees. They were mounted at a height of 25 cm above the floor to capture movement at lower-leg/shin level, which is the body region used by the algorithm for step-event detection.

The three-sensor arrangement was used to increase spatial coverage of the walking area and to reduce lower-limb occlusion during natural movement in the apartment. The sensors did not directly identify anatomical body parts. Instead, raw LiDAR distance measurements were transformed from polar coordinates into Cartesian coordinates, allowing samples from all sensors to be represented in a common metric coordinate system. The sensor data were then temporally synchronized and spatially aligned so that measurements from the three sensors could be combined into a common frame of reference.

Leg tracking was performed using a previously developed and evaluated LiDAR-based gait-analysis algorithm [24]. In brief, static environmental structures, such as walls and furniture, were separated from moving foreground samples by motion segmentation. The foreground samples were then clustered over time to identify the participant’s lower-limb movements. The resulting clusters were assigned to the left and right legs based on their spatial position and temporal continuity across consecutive frames. Gait parameters were subsequently derived from the tracked leg trajectories by calculating velocity profiles and identifying step events.

**Figure 2:**
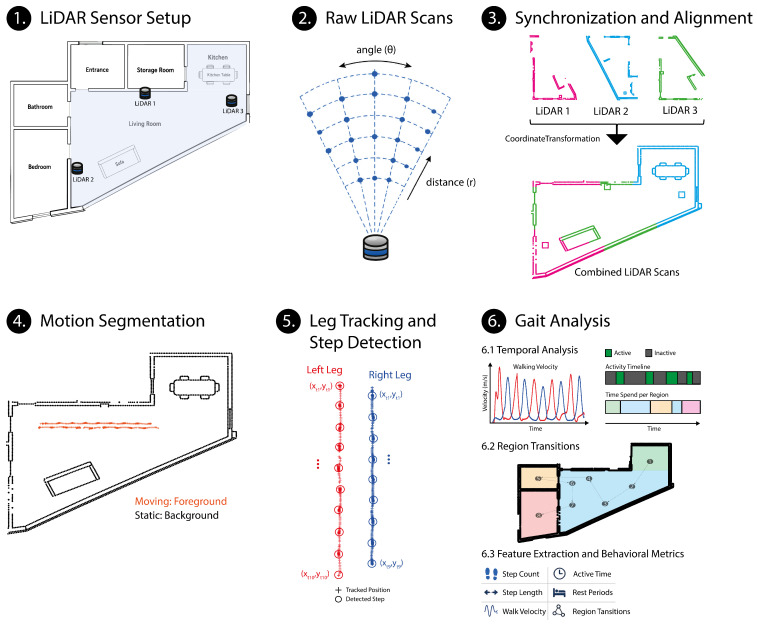
Overview of the LiDAR data-processing pipeline. (**1**) Multiple 2D LiDAR sensors are installed in the apartment to provide spatial coverage of the living room area. (**2**) Each sensor records raw scans consisting of radial distance measurements at discrete bearing angles in the sensor-local coordinate frame. (**3**) Scans from different sensors are time-synchronized, converted from polar measurements to Cartesian point coordinates, and transformed into a shared floor-plan coordinate system using sensor-specific spatial calibration. (**4**) The static background is removed to segment moving foreground points associated with human motion. (**5**) Foreground points are clustered and tracked over time to estimate left- and right-leg trajectories and identify step events during walking. (**6**) The resulting trajectories are analyzed to characterize gait and behavior, including (**6.1**) temporal activity patterns, (**6.2**) region occupancy and transitions, and (**6.3**) spatiotemporal gait and behavioral metrics such as step count, step length, walking velocity, movement-active time, rest periods, and region transitions.

Discrete lower-limb movement was therefore estimated from the position and temporal progression of moving shin-level clusters within the sensor-covered area rather than from video images or wearable sensors. This approach assumes that the monitored participant is the only moving person in the sensor-covered area. Segments with unreliable trajectories or insufficient walking activity were excluded from gait-parameter estimation. The overall processing pipeline from raw LiDAR scans to gait and activity features is summarized in Figure 2.

### 2.4. Data Processing and Feature Extraction

We linked each LiDAR position to its location on the NeuroTec Loft floor plan (Figure 1) in three steps: (1) manually dividing the floor plan into labeled areas (i.e., kitchen table, kitchen, living room, bedroom, bathroom, and sofa) to create a layout map; (2) aligning the tracked LiDAR positions to the coordinate system of this map; and (3) assigning each position to a region by checking which labeled area contained it.

For regions covered by the LiDAR setup, participant position was represented in the region-assignment time series, including sedentary periods during which no walking bout was detected. Periods without walking were therefore not treated as interpolated walking. If no movement or transition was detected at a given time point, we assumed the person remained at the previous position (e.g., in the sofa region). During sedentary periods, region occupancy was maintained from the last reliably assigned position or inferred entry–exit state; these periods were not used for gait-parameter estimation. For regions without continuous direct LiDAR coverage, specifically the bathroom and bedroom, occupancy was inferred from detected entry and exit transitions, and the corresponding duration was calculated from the interval between these transitions. This last-known-position assumption may introduce positional error if a transition occurred undetected, particularly near areas with limited direct sensor coverage. When abrupt positional jumps occurred, spatial linear interpolation was applied to maintain continuous tracking, but interpolated segments were not used for gait-parameter estimation when the underlying walking trajectory was unreliable. Where available, the frequency and duration of inferred and interpolated states were summarized as technical data-quality indicators.

We defined “movement-active time” using a 30-s rolling window: a participant was classified as movement-active at time point *t* if any walking or movement activity was detected within the interval [t−30 s, *t*]. The 30-s window was chosen a priori as a pragmatic temporal-smoothing interval, consistent with epoch- and window-based processing approaches commonly used in activity monitoring [28,29], but was not intended as a validated clinical threshold. This temporal-smoothing approach captures both sustained and brief movement episodes while filtering instantaneous noise, with the percentage of movement-active time calculated as the proportion of retained monitoring time points that meet this criterion. Because movement-active percentages depend directly on the selected window length, we performed a sensitivity analysis using alternative window lengths of 10 s and 60 s where possible (Appendix A, Table A2). Movement-active time was calculated from the timestamp-level movement-activity time series and could include detected movement episodes that were not retained for gait-parameter estimation. Valid gait duration was calculated only from walking segments that passed gait-parameter quality control; therefore, movement-active time and valid gait duration are not expected to sum to the same value. Rest periods were summarized separately from movement-active time and were not defined as the complement of movement-active time; the rest summary should therefore be interpreted as a separate descriptive rest-period summary rather than as total inactivity time.

Applying this region-assignment procedure to all time points produced a continuous measurement of region-to-region transitions over time. These transitions were defined as changes between labeled regions of the NeuroTec Loft floor plan and were used descriptively to contextualize behavior by relating region changes to the ADLs performed during the loft stay. Furthermore, we quantified the time spent in each region using the tracked position data. This quantification was visualized to provide insights into movement patterns and spatial occupancy. We also calculated ambulatory activity and spatiotemporal gait parameters and reported descriptive statistics for valid step count, step length (m), and walking velocity (m/s). Valid gait duration, valid steps, step length, walking velocity, and distance were calculated only from walking segments that passed gait-parameter quality control. Finally, step length and velocity were averaged in 30-min intervals to examine temporal variations in gait performance and compared with each individual’s region-to-region transition profile.

Unless otherwise specified, 30-min gait summaries were interpreted as unweighted interval-level descriptive estimates based only on valid gait segments retained within each interval.

Because no concurrent reference gait system was used in this proof-of-concept study, gait parameters are reported as LiDAR-derived estimates and should not be interpreted as analytically validated measurements for pwMS abnormal gait in this dataset.

### 2.5. Technical Feasibility and Data-Quality Indicators

To make the feasibility claim more transparent, we quantified post hoc technical and data-yield indicators from the existing LiDAR recordings. These indicators were used to characterize whether the system generated usable gait and activity data during the home-like monitoring session; they were not intended to validate clinical fatigue detection. The indicators included actual monitoring duration, retained LiDAR recording available for processing, region-assigned participant-present time, number of retained valid gait segments, number of valid steps contributing to each 30-min interval, and number of 30-min intervals with sufficient walking activity for gait-parameter estimation. The exported processing results contained the gait segments retained for analysis but did not include the total number of initially detected walking bouts or the number rejected during quality control. We therefore report retained valid gait segments, interval-level valid steps, and valid 30-min gait intervals as indicators of data yield. Where available, we also summarized the frequency and duration of inferred position states and interpolated segments. Walking bouts or intervals were excluded from gait-parameter estimation when the tracked lower-limb trajectory was unreliable or when walking activity was insufficient to derive stable velocity or step-length estimates. Because this was an initial proof-of-concept analysis, some frame-level quality-control labels were not prospectively prespecified; unavailable indicators are reported as not retained by the processing pipeline and are discussed as a limitation.

### 2.6. Outcome Measures and Analytical Approach

For the purpose of this exploratory analysis, we operationalized a potentially motor fatigue-relevant gait decline as a decline in mean motor performance parameters (step length or walking velocity) of ≥15% between morning and afternoon, consistent with previous research [30]. For this pilot, the threshold was used exploratorily with reference to minimal detectable change ranges reported in MS gait assessments [31], recognizing that validation in larger samples is required. This threshold was not treated as a clinically validated cutoff or diagnostic criterion for motor fatigue. Because no standardized fatigue-induction protocol, independent objective fatigue gold standard, or concurrent gait-reference measurements were included, the threshold was used only to identify candidate within-participant patterns for future validation.

For the morning–afternoon comparison, gait parameters and region-to-region transitions were summarized before and after 13:00. The 13:00 cutoff was used as a pragmatic fixed clock-time split to separate earlier from later parts of the monitored session and was applied consistently across participants to support descriptive within-participant comparison during the one-day monitoring period. However, the analyzed participant-present durations differed between participants (6.5–9.0 h), and a fixed cutoff may influence morning–afternoon summaries. Therefore, where sufficient walking data were available, we also performed a sensitivity analysis using the valid-gait-interval midpoint, defined as the midpoint between the first and last valid gait intervals, as the split. The corresponding fixed-split and valid-gait-interval midpoint values are reported in Appendix A (Table A1). Percentage changes were calculated relative to the values before the respective split.

Given the small case-study sample, missing VAS-F data for one participant, and absence of an independent fatigue ground truth, analyses were restricted to descriptive summaries (i.e., no inferential statistics, correlation analyses, ROC analysis, or high- versus low-fatigue subgroup comparisons were performed). Region-to-region transitions and activity patterns were analyzed descriptively as contextual activity measures but were not used to classify motor fatigue.

## 3. Results

### 3.1. Technical Feasibility and Data Yield

LiDAR monitoring was completed for all three participants during the scheduled home-like stay. Table 1 summarizes the available technical feasibility and data-yield indicators. The system generated continuous region-occupancy and walking-activity summaries for each participant, and valid gait estimates were obtained from retained valid gait segments across the monitoring sessions. The number of valid gait segments, valid steps, and valid gait duration contributing to each 30-min gait estimate is provided in Appendix B (Table A3).

### 3.2. Descriptive Statistics

Table 2 summarizes demographic and clinical data, as well as descriptive statistics of spatiotemporal gait parameters for the three measured individuals (P01, P02, and P03). Participant-level clinical context and exploratory morning–afternoon LiDAR-derived gait changes are summarized in Table 3.

### 3.3. Qualitative Analysis of Behavioral Profile

**Figure 3:**
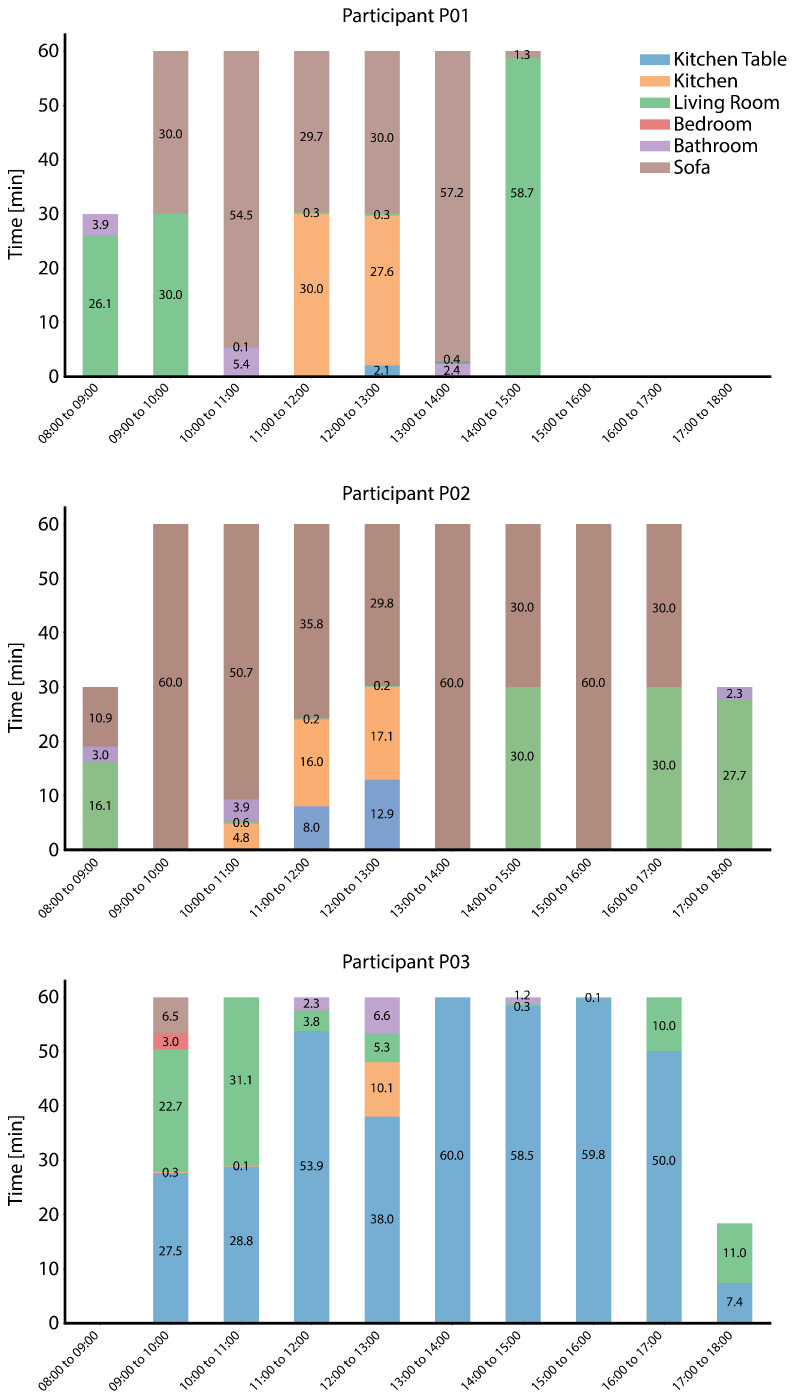
Bar plot visualizing the hourly aggregation of the time spent in each region in the NeuroTec Loft. Each region is represented by a distinct color. The x-axis shows the time intervals, while the y-axis indicates the duration (in minutes) that the participant spent in each region. The absence of a bar in the plot indicates that the participant was not present in the loft during that time interval.

Figure 3 shows the hourly aggregation of time spent per region. Participants P01 and P02 showed similar patterns regarding the distribution of time spent in different regions, whereas P03 spent most of the monitored time at the kitchen table.

P01 and P02 spent 202.5 min (52.0%) and 367.2 min (68.0%), respectively, in the sofa region; 115.9 min (29.7%) and 104.8 min (19.4%), respectively, in the living room; 57.6 min (14.8%) and 37.9 min (7.0%), respectively, in the kitchen; 2.1 min (0.5%) and 20.9 min (3.9%), respectively, at the kitchen table; and 11.7 min (3.0%) and 9.2 min (1.7%), respectively, in the bathroom. P03 spent 383.9 min (77.1%) at the kitchen table, 84.2 min (16.9%) in the living room, 10.5 min (2.1%) in the kitchen, 10.1 min (2.0%) in the bathroom, 6.5 min (1.3%) in the sofa region, and 3.0 min (0.6%) in the bedroom.

### 3.4. Progression of Gait Parameters and Transitions During the Stay

**Figure 4:**
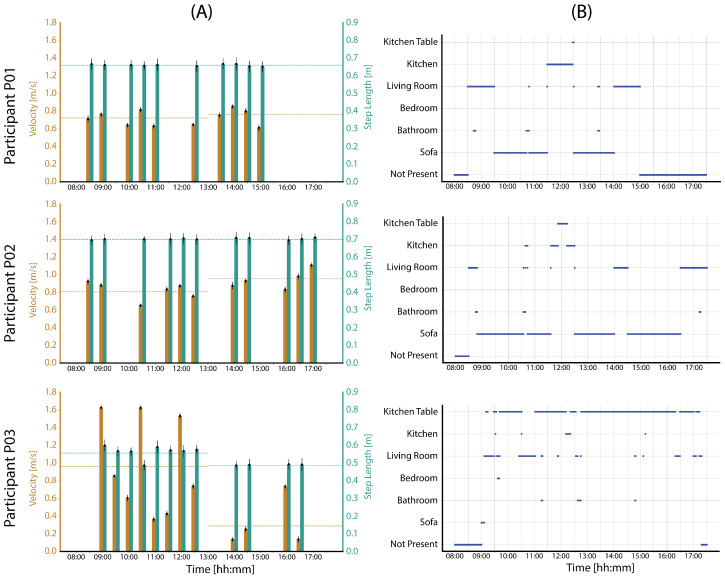
Temporal progression of gait parameters and region-transition profiles for participants during their stay in the loft. (**A**) Spatiotemporal gait parameters visualized as bar plots showing 30-min averages of LiDAR-derived walking velocity (brown) and step length (green). Black error bars indicate the variability (SD) of the measured parameter within each 30-min interval. The absence of bars indicates that no valid gait parameters were available for the corresponding interval, typically due to no walking or insufficient ambulatory activity. The number of retained valid gait segments and valid steps contributing to each 30-min interval is reported in Table A3. Dotted lines represent the average values for the fixed 13:00 split, comparing the period up to and including 13:00 with the period after 13:00 for the corresponding gait parameter. (**B**) Transition profiles depicting each participant’s movement across regions (i.e., kitchen table, kitchen, living room, bedroom, bathroom, and sofa), with blue lines indicating periods of presence in each region. The label “Not Present” signifies that the participant was not present in the loft during the corresponding time period.

Figure 4 illustrates the temporal progression of activity profiles for each participant. Panel A displays step length (green) and walking velocity (brown) statistics averaged across 30-min intervals, with error bars (black) indicating their variability (standard deviation; SD). The dotted lines indicate the average values for the fixed 13:00 split, comparing the period up to and including 13:00 with the period after 13:00. Intervals lacking gait parameters reflect periods with no walking or insufficient ambulatory activity to estimate gait parameters. Panel B depicts region transition patterns throughout the monitored session, providing spatial context for the gait variability observed in panel A.

The descriptive analysis of gait parameters showed distinct temporal patterns across the three participants (see Figure 4). P01 maintained stable values throughout the monitored session, while P02 showed stable step length and slightly increased velocity after 13:00. P03 displayed irregular step length (higher variability than P01 and P02) and a marked velocity decline after 13:00. Detailed gait statistics are listed in Table 4.

Applying the exploratory ≥15% morning–afternoon decline rule, only P03 exceeded the rule for walking velocity, with a 68.0% decrease between the two periods. P03 also showed a smaller decline in step length (14.0%), which remained below the ≥15% threshold. P01 did not exceed the rule for either parameter (velocity +7.1%; step length +1.5%), while P02 showed increased afternoon velocity and stable step length (velocity +15.9%; step length +1.4%). VAS-F scores were available for P01 and P03 only and were 1.0 and 6.5, respectively.

The qualitative interpretation of the exploratory decline rule was unchanged when using the valid-gait-interval midpoint instead of the fixed 13:00 split (Appendix A, Table A1).

Operationally defined rest periods occupied roughly one-quarter to two-fifths of each analyzed duration. P03 had no morning rest period according to this definition, whereas P01 and P02 showed rest periods distributed across the day (see Table 5). The participants completed 19 (P01: 13 in the morning, six in the afternoon), 22 (P02: 17 in the morning, five in the afternoon), and 44 (P03: 30 in the morning, 14 in the afternoon) region transitions in total, with fewer transitions after 13:00 for all the participants in this observation.

## 4. Discussion

The primary contribution of this pilot proof-of-concept study is to provide technical feasibility and data-yield evidence for the use of privacy-preserving LiDAR sensors for contact-free assessment of motor function and behavior in pwMS in a home-like setting. We monitored three pwMS with varying disability levels (EDSS scores 1–3) over analyzed participant-present durations of 6.5–9.0 h in the NeuroTec Loft while they performed self-selected ADLs, continuously recording gait and activity patterns that are potentially relevant to motor fatigue. To our knowledge, this is among the first studies to use LiDAR-based monitoring to analyze gait, activity, and region occupancy in pwMS to derive candidate digital measures for daily functioning and fatigue-relevant motor-performance changes. Importantly, the present study does not establish clinical validity, diagnostic accuracy, or fatigue specificity; rather, it provides descriptive and hypothesis-generating case-level evidence for future validation studies.

Several gait parameters differed from published reference values reported for healthy adults. While healthy adults typically walk indoors at 0.83 m/s [32], P01 (0.72 ± 0.08 m/s) and P03 (0.75 ± 0.53 m/s) showed velocities below that reference. P02 (0.88 ± 0.11 m/s) showed a velocity slightly above this reference. Moreover, P03 showed reduced step length (0.54 ± 0.04 m) compared with the average of 0.63 m for healthy individuals [33]. These observed differences are consistent with subtle performance alterations reported in MS and may be compatible with early gait-related motor changes even at low disability levels, aligning with reports of altered gait cycle timing in MS [34]. However, these comparisons provide only contextual information. Because the reference values were derived from different study populations and measurement systems and no concurrent healthy-control or gait-reference measurements were collected, these comparisons cannot establish early gait-related motor impairment or validate the LiDAR estimates in the present pwMS sample. Our findings align with accelerometer-based studies [19,35] and complement them by demonstrating the feasibility of continuous unobtrusive monitoring without requiring body-worn sensors. This convergence with previous sensor-based findings supports the plausibility of the LiDAR-derived gait patterns observed in this pilot study, while larger validation studies with concurrent gait-reference systems remain necessary. Therefore, the present data support technical feasibility and descriptive interpretability but not analytical validation of LiDAR-derived gait parameters in pwMS. LiDAR-based assessments extend beyond analyzing absolute gait parameters by capturing intraday fluctuations in gait and activity patterns that may be relevant to state fatigue (transient and situational). In contrast, characterizing trait fatigue (extended period, chronic effects) would require repeated measurements over longer observation periods. This distinction is important because trait fatigue captures the sustained impact of disease on daily functioning [36]. LiDAR measurements offer a promising method for exploring gait and activity fluctuations that are potentially relevant to motor fatigue, which are difficult to capture with momentary assessments.

The LiDAR-based region assignment distinguished specific regions (kitchen table, kitchen, living room, bedroom, bathroom, and sofa) linked to distinct ADLs (eating, cooking, resting, toileting, and leisure). Transitions between regions reflect the frequency and spatial diversity of movement behavior, providing proxy measures of mobility and ADL engagement. In pwMS, reduced transitions or prolonged stays may provide contextual information about activity organization and should be investigated further as possible indicators of fatigue-related activity changes. Hourly aggregation revealed behavioral differences among participants (Figure 3). P01 and P02 followed similar patterns, spending mornings and late afternoons in the living room/sofa areas and using the kitchen around midday. In contrast, P03 was largely sedentary at the kitchen table. P03’s kitchen table preference (77%) versus P01/P02’s sofa preference (52–68%) may represent different positioning preferences. Although one possible hypothesis is that these patterns reflect task-focused versus rest-focused positioning, the present study cannot determine whether such preferences reflect fatigue, comfort, personal preference, task choice, or the limited range of activities available in the apartment. These observed spatial patterns warrant further investigation as contextual behavioral descriptors and hypotheses for future controlled and longitudinal studies rather than as evidence of a specific fatigue-management explanation. All the participants showed predominantly sedentary routines, providing descriptive insights into activity behavior and gait changes that may inform future digital measures of fatigue-relevant motor-performance changes in MS.

These insights might help to inform future studies aiming to differentiate fatigue mechanisms, including peripheral fatigue, which may improve with rest, and central fatigue [37]. In MS, fatigue can be triggered by both physical exertion and cognitive demands, which may also contribute to concentration difficulties [37]. However, the present proof-of-concept study did not directly assess peripheral or central fatigue mechanisms. Therefore, the observed differences in spatial activity profiles should be interpreted as contextual behavioral descriptors rather than as evidence of a specific fatigue mechanism. Future protocols combining LiDAR monitoring with repeated motor-fatigability tests, perceived exertion ratings, cognitive-load measures, and patient-reported outcomes could examine whether spatial activity and gait patterns differ between fatigue mechanisms. In this context, integrating spatiotemporal region-occupancy analysis with gait metrics may help to capture real-life functional consequences of MS more comprehensively.

The behavioral profiles provide insights into daily functioning patterns that are relevant to quality of life and may not be fully captured by episodic clinical assessments. P03’s prolonged sedentary periods at the kitchen table with reduced transitions may indicate a distinct activity pattern that could be compatible with energy-conservation strategies, which are clinically relevant in MS but may be underrepresented in episodic assessments. However, the present proof-of-concept dataset cannot determine whether this pattern reflects fatigue, comfort, personal preference, task choice, or the limited range of activities available in the apartment. In future validated studies, these objective daily activity measures could help clinicians to better understand the hidden burden of MS, including situations in which patients maintain independence despite restricted activity diversity. From a physiotherapy perspective, such information may help to identify when walking performance or movement diversity decreases during the day, supporting individualized advice on pacing, rest scheduling, task distribution, and targeted gait or endurance interventions. Future studies should correlate these metrics with patient-reported outcomes, such as the MS Impact Scale [38], to establish their validity as indicators of quality-of-life-relevant daily functioning.

Comparing 30-min averages of velocity and step length (panel A) with region-to-region transitions (panel B) for each participant (Figure 4), P01 (EDSS 1.0) and P02 (EDSS 3.0) showed consistent gait profiles with stable values (Figure 4, panel A) and comparatively stable region-transition profiles (Figure 4, panel B), suggesting limited intraday variation in the selected LiDAR-derived gait summaries under the present study condition. P02’s comparatively low movement-active time (16.44%) despite having walked the greatest distance suggests that movement-active time, step count, and distance walked may capture different aspects of movement behavior. Alternatively, this finding may indicate an efficiency-over-frequency pattern; however, this remains a hypothesis for future studies rather than evidence of an adaptive fatigue-management strategy. P03 (EDSS 3.0) showed the most pronounced irregularities. This was also reflected in the transition data: a reduction in the number of region changes after 13:00 coincided with lower afternoon walking velocity in P03. This pattern may be compatible with a potentially fatigue-relevant decline in motor performance, but, because fewer transitions after 13:00 were observed in all the participants and may reflect task context, transition data were interpreted descriptively rather than as direct evidence of fatigue-related motor decline. Comparing average velocity and step length before and after the fixed 13:00 split revealed noticeable differences across the participants, with P03 showing the largest morning–afternoon changes. These observations were described alongside P03’s VAS-F score of 6.5, which is above published mean VAS fatigue scores in MS cohorts and exceeds a previously proposed VAS cutoff for high fatigue impact in people with MS [39,40]. However, the present dataset cannot establish a validated association between self-reported fatigue and LiDAR-derived gait changes, and FSMC and VAS-F were used only as descriptive clinical context rather than as interchangeable fatigue ground-truth measures. The observed intraday variations in gait parameters and activity patterns should therefore be interpreted as candidate fatigue-relevant motor-performance patterns requiring future validation. This cautious interpretation is compatible with prior accelerometer-based work suggesting that daily gait variation in MS may include deterioration in step regularity and walking velocity throughout the day [30], but the present data do not test the underlying fatigue mechanism. In addition, some 30-min-interval estimates, particularly in the P03 afternoon period, contained few valid steps and were sensitive to descriptive aggregation choices. The ability to detect such transient gait and activity fluctuations may inform future validation studies of personalized fatigue-management strategies and physiotherapy-informed activity planning aligned with individual daily patterns.

Our observation of reduced step length in the participant showing the strongest fatigue-relevant gait change aligns with previous studies [41,42]. A similar declining trend in velocity occurred between morning and afternoon, further illustrating that LiDAR-derived gait summaries can capture measurable intraday changes that may be evaluated as fatigue-relevant candidate patterns in future validation studies. All the individuals performed fewer than 1500 steps during the observation period, covering approximately 600–770 m. These values are consistent with low within-session ambulatory activity during the loft stay and, for context only, fall below step-based low-activity thresholds proposed for full-day monitoring, where fewer than 2500 steps/day have been proposed as low activity [43]. However, because monitoring was limited to analyzed participant-present durations of 6.5–9.0 h in a constrained home-like apartment and depended on the optional activities selected by each participant, these values should not be interpreted as habitual daily step counts, classified as full-day low activity, or directly compared with full-day free-living activity categories or physical-activity recommendations. This interpretation is supported by the low movement-active time recorded during the monitored session (Table 2). Taken together with the observed reductions in velocity and step length in one participant, these session-level activity summaries may provide candidate variables for future studies of fatigue-relevant motor-performance changes, but they do not establish fatigue-specific or clinical activity markers. Although the present protocol does not directly assess habitual free-living mobility, even relatively short bouts of walking in a home-like setting can provide quantifiable information on individual gait and activity patterns in pwMS. General physical-activity recommendations [44] provide an important reference for future free-living validation studies, but the present single-session data cannot be used to assess adherence to such recommendations. This underscores the potential value of LiDAR-based monitoring as a candidate digital measure to characterize gait and activity changes in MS and highlights its possible future role, if validated in larger longitudinal and controlled studies, in informing timely targeted therapeutic interventions before more pronounced disability develops.

Despite the observed differences in gait parameters between morning and afternoon, as well as the overall low activity level during the stay (LiDAR-derived movement-active time, defined using a 30-s rolling window, accounted for only 16–32% of monitored time), our findings should be interpreted with caution for several reasons. Sedentary tasks, such as working on a notebook or watching a film, might provide an alternative explanation for the reduced activity. The apartment environment might also have provided limited stimulation, thereby reducing the motivation to engage in physical activity. In addition, the single-day analyzed monitoring period of 6.5–9.0 h might fail to capture typical daily patterns or habitual free-living activity.

The small sample size (n=3) and the single-day observation limit the generalizability of our findings and preclude inferential statistics, subgroup comparisons, diagnostic cutoff validation, or clinical validation of fatigue-related outcomes. No healthy control participant group or stratified MS comparison group was included under the same loft protocol. Therefore, we cannot rule out that reduced activity levels or morning–afternoon changes were partly attributable to the experimental environment, task selection, age, individual habits, or other contextual factors rather than to MS-related motor fatigue.

No standardized fatigue-induction protocol or independent reference standard for motor fatigue was included, and VAS-F data were unavailable for one participant. FSMC and available VAS-F scores were therefore treated as descriptive clinical context rather than objective motor-fatigue ground-truth measures. No concurrent gait-reference system, such as GAITRite, optical motion capture, or wearable IMUs, was recorded during the NeuroTec Loft stay; therefore, the present dataset does not provide analytical validation of LiDAR-derived gait estimates in pwMS. Although the LiDAR gait-analysis algorithm was developed and evaluated in previous work [24], the present dataset did not include concurrent reference-system measurements in the same pwMS participants. Therefore, this study cannot establish the measurement accuracy of LiDAR-derived gait parameters for abnormal gait in pwMS. Controlled conditions with concurrent reference measurements and standardized fatigue assessments are required to reduce confounding and support reliable interpretation of fatigue-related motor changes in MS. Consequently, the observed morning–afternoon changes should be interpreted as candidate fatigue-relevant motor-performance patterns requiring future validation, not as validated fatigue markers. In addition, some interval-level gait estimates, particularly for P03, showed high variability and may have been influenced by short walking bouts, turning, constrained-space movement, or residual tracking artifacts that could not be adjudicated without concurrent reference measurements.

Region occupancy in the bathroom and bedroom was inferred from entry–exit transitions, and last-known-position continuity was used during periods without detected movement. The available post hoc indicators summarize time in non-directly covered regions and interpolated walking segments, but the original processing pipeline did not retain all the frame-level labels needed to quantify every possible last-known-position error. In addition, because the original proof-of-concept processing export retained the gait segments used for analysis but not the total number of initially detected candidate walking bouts, we could not quantify the proportion of candidate bouts rejected during quality control. Future versions of the pipeline should retain these intermediate labels prospectively to support more complete tracking-success and exclusion-rate reporting. Region-transition counts may also be sensitive to manually defined region boundaries and short boundary-adjacent movements. Additional participant-level factors such as height, medication status, recent activity, sleep, heat sensitivity, and MS phenotype were not systematically analyzed and may have influenced gait and activity patterns.

Our contribution lies in simultaneously capturing both fine-grained gait measures (step-level gait) and broader behavioral measures (region transitions and activity patterns) using privacy-preserving contact-free technology. These findings provide preliminary feasibility evidence of using LiDAR sensors to derive privacy-preserving digital measures of gait and activity in MS. They also warrant multicenter validation studies to determine whether clinically meaningful thresholds can be established. Additionally, future longitudinal studies should investigate whether LiDAR-derived gait and activity parameters have predictive value for disease progression or fatigue-related functional change, and whether feedback based on these parameters could support individualized monitoring or management of motor fatigue.

## 5. Conclusions

This exploratory pilot study demonstrates the technical feasibility of using LiDAR-based monitoring to continuously capture gait and activity parameters in pwMS during a one-day stay in a home-like environment. The observed individual differences in walking velocity, step length, region transitions, and sedentary behavior suggest that LiDAR-derived parameters may serve as candidate digital measures for characterizing daily functioning and fatigue-relevant changes in motor performance. In particular, one participant exceeded the exploratory decline criterion for afternoon walking velocity, whereas the step-length decrease remained below the criterion. However, the small sample size, single-day observation, absence of a healthy control group, lack of concurrent gait-reference measurements, and absence of an independent gold standard or standardized induction protocol for fatigue preclude conclusions about clinical validity, fatigue specificity, diagnostic thresholds, or diagnostic performance. Larger longitudinal and controlled studies with concurrent reference systems and standardized fatigue assessments are required before LiDAR-derived gait and activity measures can support clinical or fatigue-specific interpretation. Such studies should determine whether these measures can reliably capture fatigue-relevant motor-performance changes, whether clinically meaningful thresholds can be established, and whether they can contribute to individualized fatigue management in MS.

## Figures and Tables

**Table 1:** Technical feasibility and data-yield indicators derived from the LiDAR recordings. Nominal scheduled duration refers to the planned participant-observation duration. Analyzed region-assigned participant duration refers to the participant-present time represented in the region-assignment and activity summaries. Analyzed participant duration could differ from the nominal scheduled duration because it was based on the actual participant-present interval represented in the processed region-assignment data. Retained LiDAR recording duration additionally included protocol buffer periods. Retained LiDAR recording available for processing was calculated relative to the retained recording duration and capped at 100%. Region-assigned participant-present time refers to the proportion of analyzed participant-present time assigned to a region label. Time in directly LiDAR-covered regions refers to regions with direct LiDAR coverage, whereas time inferred in non-directly covered regions refers to bathroom and bedroom occupancy inferred from entry–exit transitions. Interpolated walking segments refer only to walking trajectories used for gait-parameter estimation; no interpolated walking trajectories were included. The table reports retained valid gait segments. The total number of initially detected walking bouts and the number rejected during quality control were not available in the exported processing results.

	P01	P02	P03
Nominal scheduled participant-observation duration (h)	7.0	9.0	8.0
Analyzed region-assigned participant duration (h)	6.5	9.0	8.3
Retained LiDAR recording duration (h)	7.5	9.5	8.5
Retained LiDAR recording available for processing (%)	100.0	100.0	100.0
Region-assigned participant-present time (%)	100.0	100.0	100.0
Time in directly LiDAR-covered regions (%)	97.0	98.3	97.4
Time inferred in non-directly covered regions (%)	3.0	1.7	2.6
Interpolated walking segments (n)	0	0	0
Total interpolated walking duration (s)	0.0	0.0	0.0
Retained valid gait segments (n)	19	22	44
30-min intervals with valid gait estimates (n)	10	11	12
Median valid steps per valid 30-min interval	101.5	90.0	78.0
Minimum–maximum valid steps per valid 30-min interval	28–200	26–250	7–269

**Table 2:** Average values of the spatiotemporal gait parameters recorded during the observation period, as well as demographic and clinical assessment data. Note. Values represent means with standard deviations in parentheses for continuous variables. Distance walked represents the measured total walking distance. N/A = not applicable. EDSS = Expanded Disability Status Scale; FSMC = Fatigue Scale for Motor and Cognitive Functions; VAS-F = Visual Analogue Scale for Fatigue. VAS-F data for P02 were not collected due to time constraints during the clinical assessment session.

	P01	P02	P03
Age (years)	30	63	26
Sex	female	male	female
Months since diagnosis	2	6	4
EDSS	1	3	3
FSMC	64	73	73
VAS-F	1	N/A	6.5
Analyzed duration (h)	6.5	9.0	8.3
Movement-active time (%)	32.00	16.44	29.50
Velocity (m/s)	0.72 (0.08)	0.88 (0.11)	0.75 (0.53)
Step length (m)	0.66 (0.01)	0.70 (<0.01)	0.54 (0.04)
Steps (n)	1004	1096	1124
Distance walked (m)	663	767	606

**Table 3:** Participant-level clinical context and exploratory morning–afternoon LiDAR-derived gait changes. Values are descriptive case-level summaries only and were not used for inferential testing. VAS-F was unavailable for P02.

	P01	P02	P03
EDSS	1	3	3
FSMC	64	73	73
VAS-F	1	N/A	6.5
Velocity change after 13:00	+7.1%	+15.9%	−68.0%
Step-length change after 13:00	+1.5%	+1.4%	−14.0%
Total steps during monitored stay	1004	1096	1124
Total region transitions	19	22	44
Morning region transitions	13	17	30
Afternoon region transitions	6	5	14
Exploratory ≥15% decline rule exceeded	No	No	Velocity only

**Table 4:** Aggregated LiDAR-derived spatiotemporal gait parameter statistics by participant. Overall values summarize retained valid gait segments across the full measurement session. Before and after 13:00 rows summarize the period-level gait estimates used for the exploratory fixed 13:00 comparison. Values are *M* (SD); Δ represents the change after 13:00 minus before 13:00. The appendix sensitivity analysis reports interval-level summaries; small numerical differences may occur because different aggregation levels were used. Sparse intervals should be interpreted cautiously. Values are descriptive case-level summaries and were not used for inferential testing.

	P01	P02	P03
Velocity (m/s)			
Overall	0.72 (0.08)	0.88 (0.11)	0.75 (0.53)
Before 13:00	0.70 (0.10)	0.82 (0.10)	0.97 (0.50)
After 13:00	0.75 (0.09)	0.95 (0.10)	0.31 (0.25)
Δ	0.05	0.13	−0.66
Step length (m)			
Overall	0.66 (0.01)	0.70 (<0.01)	0.54 (0.04)
Before 13:00	0.66 (<0.01)	0.70 (<0.01)	0.57 (0.03)
After 13:00	0.66 (0.01)	0.71 (0.01)	0.49 (<0.01)
Δ	0.01	0.01	−0.08

**Table 5:** Operationally defined rest-period statistics and resting patterns. Rest periods occupied roughly one-quarter to two-fifths of each analyzed duration. Participants P01 and P02 showed rest periods distributed across the day, while Participant P03 did not show a morning rest period according to this definition. Note. Rest percentages were calculated relative to the analyzed duration. M = mean; SD = standard deviation; Max = maximum.

	P01	P02	P03
Rest (% of analyzed duration)	30.8	38.9	24.1
Mean (min)	40.0	52.5	60.0
SD (min)	17.3	15.0	0.0
Max (min)	60.0	60.0	60.0
Pattern	Distributed	Distributed	No morning rest period

## Data Availability

The raw data that support the findings of this study are not openly available because they contain sensitive indoor movement patterns. The associated processed raw data is available from the corresponding author, Michael Single, upon reasonable request.

## Appendix A. Sensitivity Analyses

This appendix reports descriptive sensitivity analyses for the fixed 13:00 morning–afternoon split and the movement-active-time rolling-window definition.

**Table A1:** Sensitivity analysis comparing the fixed 13:00 split with a valid-gait-interval midpoint split. The valid-gait-interval midpoint was defined as the midpoint between the first and last valid gait intervals available for each participant. First and second refer to the mean values before and after the respective split point. Absolute change was calculated as second minus first, and percentage change was calculated relative to the first value. Overall interval means were calculated as weighted means according to the number of valid gait intervals before and after the split. Minor discrepancies between displayed means and changes may occur because changes were calculated from unrounded values. The qualitative interpretation refers to whether the participant exceeded the exploratory ≥15% morning–afternoon decline rule. The analysis is descriptive only.

	P01	P02	P03
Fixed 13:00 split			
Valid gait intervals, first/second half (n)	6/4	6/5	8/4
Velocity, first half mean (m/s)	0.6920	0.8162	0.9690
Velocity, second half mean (m/s)	0.7410	0.9460	0.3100
Velocity, absolute change (m/s)	+0.0490	+0.1298	−0.6590
Velocity change (%)	+7.08	+15.90	−68.01
Velocity, overall interval mean (m/s)	0.7116	0.8752	0.7493
Step length, first half mean (m)	0.6550	0.7001	0.5700
Step length, second half mean (m)	0.6648	0.7097	0.4900
Step length, absolute change (m)	+0.0098	+0.0096	−0.0800
Step length change (%)	+1.50	+1.37	−14.04
Step length, overall interval mean (m)	0.6589	0.7045	0.5433
Valid-gait-interval midpoint split			
Valid-gait-interval midpoint	11:45	12:45	12:45
First–last valid gait interval	08:30–15:00	08:30–17:00	09:00–16:30
Valid gait intervals, first/second half (n)	5/5	6/5	8/4
Velocity, first half mean (m/s)	0.7024	0.8162	0.9690
Velocity, second half mean (m/s)	0.7208	0.9460	0.3100
Velocity, absolute change (m/s)	+0.0183	+0.1298	−0.6590
Velocity change (%)	+2.61	+15.90	−68.01
Velocity, overall interval mean (m/s)	0.7116	0.8752	0.7493
Step length, first half mean (m)	0.6561	0.7001	0.5700
Step length, second half mean (m)	0.6618	0.7097	0.4900
Step length, absolute change (m)	+0.0057	+0.0096	−0.0800
Step length change (%)	+0.87	+1.37	−14.04
Step length, overall interval mean (m)	0.6590	0.7045	0.5433
Qualitative interpretation changed?	No	No	No

The sensitivity analysis using valid-gait-interval midpoint splits yielded the same qualitative interpretation as the fixed 13:00 split: P01 and P02 did not exceed the exploratory morning–afternoon decline rule, whereas P03 exceeded the rule for walking velocity only. For P01, the magnitude of the observed changes was smaller when using the valid-gait-interval midpoint. For P02 and P03, the valid-gait-interval midpoint split resulted in the same allocation of valid gait intervals as the fixed 13:00 split and therefore produced identical values. Because of the case-study design, this analysis was interpreted descriptively only.

Movement-active time percentages increased slightly with longer rolling-window lengths across all the participants (Table A2). The relative ordering of participants remained unchanged across window lengths. The 30 s window was retained for the primary analysis because it provided temporal smoothing of brief walking episodes while limiting overextension of activity labels into prolonged sedentary periods. The movement-active time values are interpreted as window-dependent descriptors of session activity, not as validated activity classifications.

**Table A2:** Sensitivity of LiDAR-derived movement-active time estimates to the rolling-window length. Movement-active time was recomputed from the same walking-activity time series using 10 s, 30 s, and 60 s windows, with a time point classified as movement-active when walking or movement activity was detected within the preceding window. The 30 s window was used as the primary analysis. Values indicate the percentage of monitored time classified as movement-active and should be interpreted as window-dependent descriptors of session activity rather than validated clinical activity classifications.

Rolling-Window Length	P01	P02	P03
10 s movement-active time (%)	30.97	15.46	27.26
30 s movement-active time (%)	32.00	16.44	29.50
60 s movement-active time (%)	33.41	17.83	32.75
Qualitative ranking changed?	No		

## Appendix B. Interval-Level Gait Data Yield

This appendix reports interval-level data-yield information for the 30-min gait estimates used to support interpretation of the temporal gait summaries.

To support interpretation of the 30-min gait estimates shown in Figure 4, Table A3 reports the number of retained valid gait segments, valid steps, and walking duration contributing to each interval. Intervals without valid gait estimates generally corresponded to periods with no walking or insufficient walking activity for reliable gait-parameter extraction.

**Table A3:** Movement-active time and valid gait estimates in 30-min intervals. The time label denotes the corresponding 30-min time interval. The first or last interval may be shorter than 30 min if the analyzed participant-present period did not cover the full interval; such intervals should not be interpreted as additional participant-observation time. The 30 s active contribution represents the interval-level contribution to the rolling-window movement-active time estimate and is not equivalent to valid gait duration. Valid gait duration, steps, velocity, and step length refer only to walking segments retained for gait parameter estimation. Dashes indicate that no valid gait estimate was available for the corresponding interval.

Participant	Time	30 s Active Contrib. (s)	Valid Gait Segments (n)	Valid Steps (n)	Valid Gait Duration (s)	Velocity (m/s)	Step Length (m)
P01	08:30	950	3	200	187.7	0.7028	0.6597
P01	09:00	800	2	158	137.3	0.7548	0.6557
P01	09:30	550	–	–	–	–	–
P01	10:00	250	1	45	46.7	0.6318	0.6557
P01	10:30	350	4	108	87.7	0.8028	0.6517
P01	11:00	760	2	95	100.8	0.6198	0.6577
P01	11:30	620	–	–	–	–	–
P01	12:00	400	–	–	–	–	–
P01	12:30	560	1	65	66.0	0.6398	0.6497
P01	13:00	200	–	–	–	–	–
P01	13:30	220	1	30	27.3	0.7390	0.6723
P01	14:00	900	2	135	108.3	0.8380	0.6723
P01	14:30	850	2	140	117.1	0.7870	0.6583
P01	15:00	74	1	28	30.6	0.6000	0.6563
P02	08:30	550	4	250	188.1	0.9232	0.6948
P02	09:00	80	1	31	24.8	0.8752	0.7008
P02	09:30	40	–	–	–	–	–
P02	10:00	220	–	–	–	–	–
P02	10:30	500	6	90	97.3	0.6482	0.7008
P02	11:00	300	–	–	–	–	–
P02	11:30	600	3	155	130.7	0.8312	0.7008
P02	12:00	650	2	118	95.9	0.8672	0.7048
P02	12:30	500	1	34	31.6	0.7522	0.6988
P02	13:00	100	–	–	–	–	–
P02	13:30	100	–	–	–	–	–
P02	14:00	600	1	80	65.2	0.8758	0.7133
P02	14:30	150	1	29	22.2	0.9318	0.7133
P02	15:00	50	–	–	–	–	–
P02	15:30	50	–	–	–	–	–
P02	16:00	300	1	26	21.9	0.8318	0.6993
P02	16:30	420	1	161	115.8	0.9838	0.7073
P02	17:00	117	1	122	78.8	1.1068	0.7153
P02	17:30	–	–	–	–	–	–
P03	09:00	550	4	99	36.8	1.6254	0.6035
P03	09:30	600	6	139	93.2	0.8524	0.5715
P03	10:00	600	3	78	74.0	0.6014	0.5705
P03	10:30	700	3	269	81.7	1.6214	0.4925
P03	11:00	450	5	63	104.7	0.3584	0.5955
P03	11:30	550	2	39	52.6	0.4264	0.5755
P03	12:00	650	3	101	37.7	1.5294	0.5715
P03	12:30	600	4	78	61.3	0.7374	0.5795
P03	13:00	450	–	–	–	–	–
P03	13:30	500	–	–	–	–	–
P03	14:00	400	1	7	26.3	0.1295	0.4865
P03	14:30	450	5	20	40.0	0.2455	0.4905
P03	15:00	350	–	–	–	–	–
P03	15:30	350	–	–	–	–	–
P03	16:00	750	6	216	145.4	0.7315	0.4925
P03	16:30	550	2	15	55.1	0.1335	0.4905
P03	17:00	318	–	–	–	–	–
